# Supplementary material for: Quadrupolar excitons in MoSe2 bilayers
Source: Nat Commun. 2025 Feb 5;16:1382. doi: 10.1038/s41467-025-56586-3 (PMC11799382; doi:10.1038/s41467-025-56586-3)
Supplement: Supplementary file 1 — Supplementary Information [file 41467_2025_56586_MOESM1_ESM.pdf]

Supplementary Information: Quadrupolar Excitons in $MoSe_2$ Bilayers

Jakub Jasiński,[1,2] Joakim Hagel,[3] Samuel Brem,[4] Edith Wietek,[5] Takashi Taniguchi,[6] Kenji Watanabe,[7] Alexey Chernikov,[5] Nicolas Bruyant,[2] Mateusz Dyksik,[1] Alessandro Surrente,[1] Michał Baranowski,[1] Duncan K. Maude,[2] Ermin Malic,[4] and Paulina Płochocka[1,2,*]

[1]*Department of Experimental Physics, Faculty of Fundamental Problems of Technology, Wroclaw University of Science and Technology, 50-370 Wroclaw, Poland*

[2]*Laboratoire National des Champs Magnétiques Intenses, EMFL, CNRS UPR 3228, Université Grenoble Alpes, Université Toulouse, Université Toulouse 3, INSA-T, Grenoble and Toulouse, France*

[3]*Department of Physics, Chalmers University of Technology, 412 96 Gothenburg, Sweden*

[4]*Department of Physics, Philipps-Universität Marburg, Renthof 7 35032 Marburg, Germany*

[5]*Institute of Applied Physics and Würzburg-Dresden Cluster of Excellence ct.qmat, Technische Universität Dresden, 01062 Dresden, Germany*

[6]*International Center for Materials Nanoarchitectonics, National Institute for Materials Science, Tsukuba, Ibaraki 305-004, Japan*

[7]*Research Center for Functional Materials, National Institute for Materials Science, Tsukuba, Ibaraki 305-004, Japan*

(Dated: January 5, 2025)

I. SUPPORTING MEASUREMENTS

Fig. S1 shows the electric field dependence of the static electric dipole moment of the Q_1, Q_2 and the $IX^A_{1s\uparrow\downarrow}$ (IX), calculated as dE/dE_z. The non-linear dependence of the quadrupolar exciton energy on the electric field translates to a vanishingly small electric dipole at low fields. The static dipole moment increases with increasing electric field and steadily approaches the dipole moment obtained for the dipolar spin-triplet interlayer exciton $IX^A_{1s\uparrow\downarrow}$.However, around 0.1 V/nm, the quadrupolar state begins to interact with other excitonic states due to the avoided crossing with A_{1s} excitons, causing the dipole moments of the quadrupolar states to deviate from predictions based on a simple two-level model.

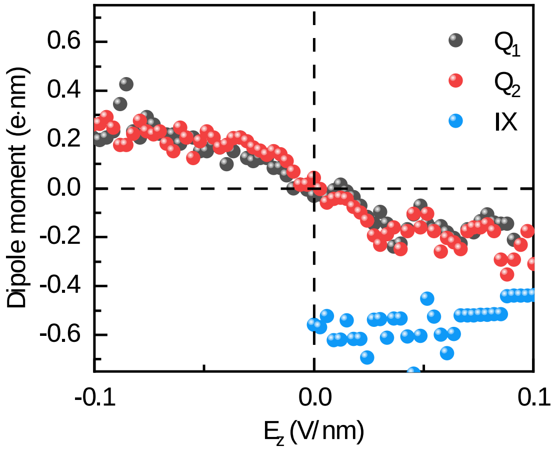

FIG. S1. **Electric field dependence of the static electric dipole moment.** Q_1 and Q_2 correspond to quadrupolar excitons and IX to the redshifting branch of spin-triplet interlayer exciton $IX^A_{1s\uparrow\downarrow}$.

We show in Fig. S2 additional reflectivity spectra as a function of the electric field E_z at two different spots (panels (a) and (b)). In all the investigated spots, we find evidence of the presence of quadrupolar excitons, as highlighted in Fig. S2. The consistent observation of these features in multiple spots on one sample, as well as their presence on another sample (see Fig. S3), strongly suggests that the quadrupolar exciton complexes are an inherent feature of natural $MoSe_2$ bilayers.

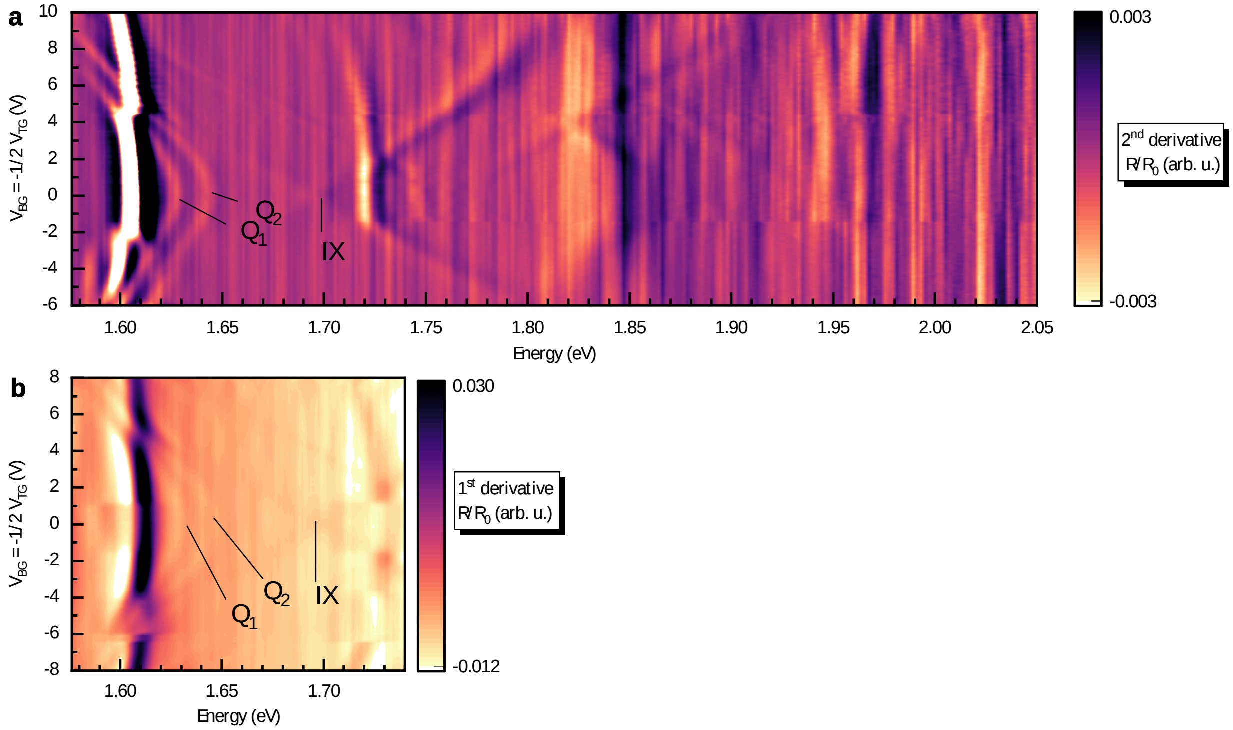

FIG. S2. **Reflectivity as a function of the electric field measured at different spots. a** and **b** False-color maps of two additional series of reflectivity spectrum measured as a function of E_z on the main device. Reflectivity shown in the form of 2nd derivative R/R_0 in **a** and 1st derivative R/R_0 in **b**. The Q_1 and Q_2 branches are marked, as well as the above dipolar interlayer exciton IX.

Fig. S3 shows false-color map of 2nd derivative of reflectivity R/R_0 as a function of electric field (opposite voltage polarity applied to bottom and top gates) measured on a second natural $MoSe_2$ bilayer device. Similar to the device shown in the main text, here also two quadratically shifting quadrupolar branches Q_1 and Q_2, as well as linearly shifting spin-triplet IXs can be observed.

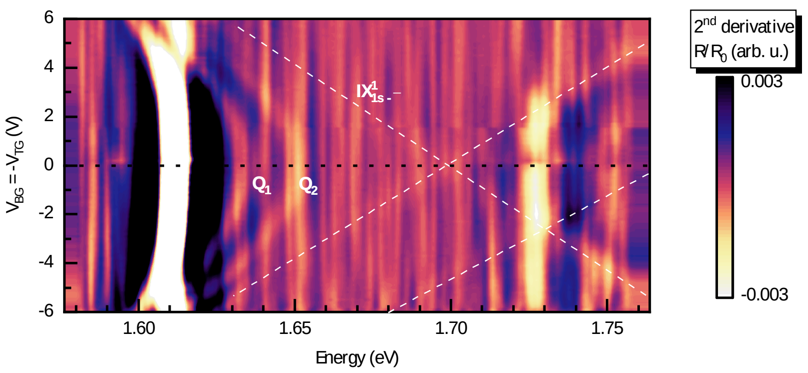

FIG. S3. **False-color map of 2nd derivative of reflectivity as a function of the out of plane electric field on the second natural $MoSe_2$ bilayer device.** Two quadrupolar branches Q_1 and Q_2 as well as the linearly shifting triplet IX states can be observed, similar as on the device shown in main text.

Fig. S4 shows the evolution of the excitonic energy landscape in a broad spectral and electric field (E_z) range. Panel (a) shows the reflectivity spectrum at zero electric field, where various resonances related to intra- and interlayer transitions are marked. Panel (b) shows the 2nd derivative of reflectivity R/R_0 as a function of the applied E_z. Aside from the quadratically shifting quadrupolar Q_1 and Q_2 excitons, several interesting features can be noted: (i) The avoided crossing and transfer of the oscillator strength between intralayer and interlayer transitions related to the hole tunneling, as in the case of the very strong mixing of the $IX'^A_{1s\uparrow\downarrow}$ and B'_{1s}. (ii) The electron tunneling related anti-crossing between the A_{1s} excitons and the Q_1, Q_2 and $IX^A_{1s\uparrow\downarrow}$ at $E_z \sim 0.1 - 0.15$ V/nm as well as with higher lying 2s IXs at higher E_z. (iii) At $E_z \sim 0.1 - 0.15$ V/nm we observe transfer of the oscillator strength between the spin-singlet $IX^A_{2s\uparrow\uparrow}$ and lower energy state (detuned by approximately 20 meV) likely related to brightened spin-triplet $IX'^A_{2s\uparrow\downarrow}$. Panel (c) shows the calculated excitonic landscape under electric field, which includes all three considered couplings. Here we can observe the very good qualitative agreement with the experimental results shown in panel (b). Particularly, the emergence of the anti-crossing at the A_{1s} exciton corroborating the experimental findings as well as several examples of hole tunneling related hybridizations (marked by purple arrows), which are also visible in the experiment. The aforementioned 2s spin-triplet state $IX'^A_{2s\uparrow\downarrow}$, the emergence of which can be observed in the reflectivity at high E_z, does not appear in the simulated spectra in panel (c) because these 2s spin-triplet states are not considered in the model. This is because they are not essential to understanding the formation of quadrupolar excitons, which are the focus of this study.

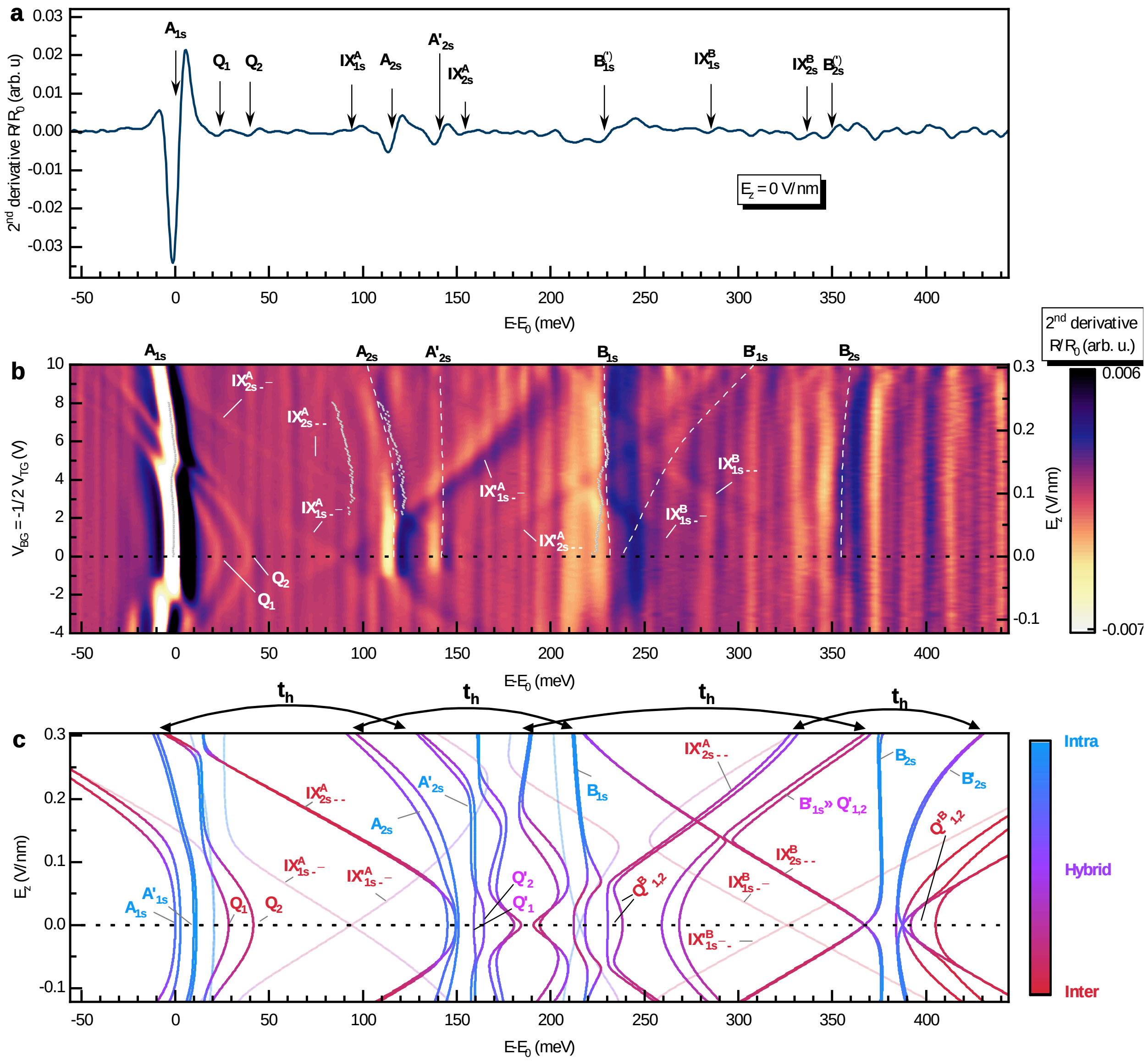

FIG. S4. **Measured and simulated exciton energy landscape under electric field. a** 2nd derivative of reflectivity at $E_z = 0\,\mathrm{V/nm}$. **b** False-color map of 2nd derivative of reflectivity as a function of the out of plane electric field. The overlaid grey points (A_{1s}, A_{2s}, B_{1s}) are taken from the fitting of the PL spectrum shown in Fig. S5. In panels **a** and **b** the energy is measured relative to the A_{1s} exciton at $E_0 = 1.606\,\mathrm{eV}$. **c** Simulation of the excitonic landscape as a function of the out of plane electric field. The opaque and semi-transparent lines correspond to singlet and triplet states, respectively. The additional superscripts “A” and “B” denote the lower A-like and higher B-like interlayer states, respectively, exemplified in Fig. S7(d).

Fig. S5 shows the photoluminescence (PL) spectrum as a function of the applied electric field in the form of a false-color map (panel (a)) and stacked individual spectra (panel (b)). At $E_z = 0\,\mathrm{V/nm}$, the strongest PL peak originates from the A_{1s} transition. On the low energy side of A_{1s}, the less intense negatively trion (T) emission is observed. At low energy side ($\sim -170\,\mathrm{meV}$ below A_{1s}) a weak, momentum-indirect interlayer exciton (IX_{ind}) can be observed. For increasing E_z (positive direction) we observe linear red shift of the aforementioned IX_{ind}, the slope of which is approximately half of the one characterizing the momentum-direct IXs visible in the reflectivity map of Fig. S4(b). This is due to the IX_{ind}, which stems from the $K - \Gamma$ transition,[1–3] where the hole at Γ point is delocalized between the two layers, effectively lowering the dipole moment. Moreover, increasing E_z results in the enhancement of the PL intensity of the A_{2s} and B_{1s} states. Interestingly, a lower energy side peak to the main A_{2s} peak appears, which most likely originates from the negatively charged A_{2s}^- trion, similar to what was observed in monolayer WSe_2.[4] At the same time, the low energy side peak of A_{1s}, attributed to trion (T) at $E_z = 0\,\mathrm{V/nm}$ also gains intensity in the same E_z range. The presence of charged exciton features in the PL spectrum points to a small degree of unintentional doping, as the reflectivity lacks these signatures. Furthermore, the PL peak energies shift rapidly, due to anti-crossings with otherwise non-emissive interlayer dipolar or quadrupolar excitons. This behaviour is visible at $E_z \sim 0.1$ – 0.15 V/nm for both A_{1s} and B_{1s} excitons, which corresponds to the electric field range where avoided crossings for these excitonic species are observed in the reflectivity spectrum of Fig. S4(b), where PL peak energies are also overlaid (grey points).

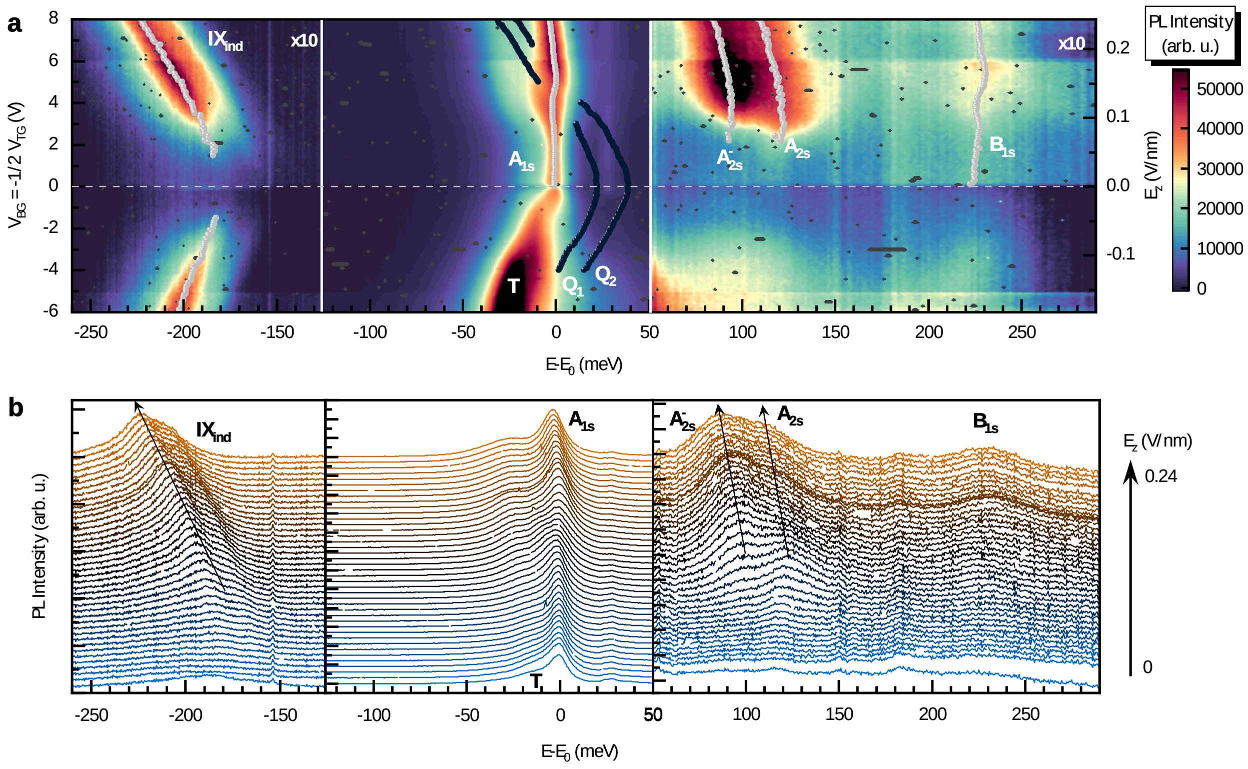

FIG. S5. **Photoluminescence responce under the electric field.** **a** False-color map of PL as a function of the out of plane electric field E_z. The overlaid black data points (Q_1 and Q_2) correspond to the energy position extracted from the Reflectivity dataset shown in Fig. S4. The overlaid grey points (A_{1s}, A_{2s}, B_{1s}) are taken from the fitting of the PL emission shown here. The quadrupolar transitions Q_1 and Q_2 are taken as the minima in the reflectivity spectrum shown in Fig. S4(b). We note here that the application of the gate voltages is not symmetrical and for the negative E_z field direction the sample shows significant doping, thus we focus on the positive E_z part. **b** Waterfall plot of the PL spectrum from E_z=0 V/nm to E_z = 0.24 V/nm. Spectra are shifted vertically for clarity. The energy scale (E-E$_0$) is shown relative to the A_{1s} exciton at $E_0 = 1.606$ eV.

Fig. S6 shows the PL spectrum (panel (a)) and 1st derivative of the reflectivity R/R_0 (panel (b)) as a function of the gate voltages applied with the same polarity, which enables free carrier doping, without simultaneous application of electric field. The gate voltage ratio accounting for the unequal thickness of bottom and top hBNs was chosen the same (in absolute value) as for the electric field sweep, i.e., $\mathrm{V}_{BG} = \frac{1}{2}V_{TG}$. As can be observed in the shown figures, the minimal carrier doping, as observed by smallest contribution of charged excitonic states (attractive Fermi-polarons[5]) as well as most prominent presence of neutral excitonic states (repulsive Fermi-polarons[5]), is in the vicinity of $\mathrm{V}_{BG} = \frac{1}{2}V_{TG} = 0$ V. For positive gate voltages, direction we can observe the change of the regime into electron doped regime, as attested by the emergence of the negatively charged A_{1s} exciton feature, which shifts away from the neutral exciton due to increasing doping level. Consequently, the negative gate voltages polarity changes the doping regime to the hole doping, as seen by the emergence of positively charged exciton feature.

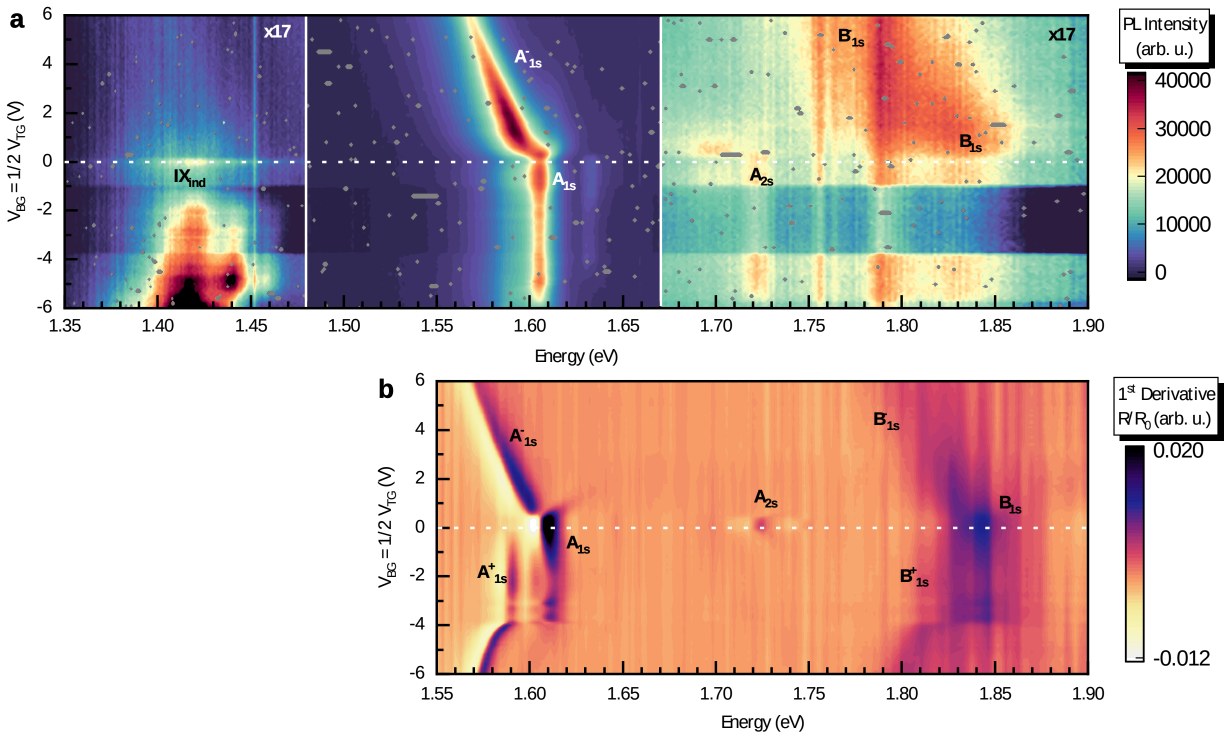

FIG. S6. **Free carrier doping dependent photoluminescence and reflectivity.** False-color map of **a** the PL spectrum and of the **b** 1st derivative of reflectivity as a function of the gate voltages (with constant ratio $V_{BG} = \frac{1}{2}V_{TG}$) corresponding to the change in free carrier concentration. Both PL and reflectivity spectra show a clear switching between different doping regimes. At $V_{BG} = \frac{1}{2}V_{TG} = 0V$, we see the most neutral regime due to the highest absorption strength of the neutral complexes of A_{1s}, A_{2s} and B_{1s} with minimized contribution of charged complexes. Thus those voltages were chosen as an optimal starting point for the electric field sweep.

II. THEORETICAL MODEL

In order to form quadrupole excitons we need a coupling between differently oriented dipoles. In its simplest form such a Hamiltonian can be written as

$$H = \begin{pmatrix} E_{IX} + d_L E_z & J \\ J^* & E_{IX'} - d_L E_z, \end{pmatrix} \tag{1}$$

where E_{IX} is the interlayer exciton energy of one dipole and $E_{IX'}$ is the energy of the reversed dipole. The linear energetic shift with the electrical field is described by $d_L E_z$, where d_L is the dipole length and E_z is the field strength. J is the coupling between the interlayer excitons. Diagonalizing such a Hamiltonian yields two quadrupole excitons with opposite curvature which we call Q and Q', and they will be energetically separated by the energy $2J$.

Starting with the single particle picture, terms that could form quadrupole excitons will emerge from the Coulomb interaction,

$$H_C = \frac{1}{2}\sum_{1234} \bar{V}^{1234} a_1^\dagger a_2^\dagger a_3 a_4, \tag{2}$$

where 1 - 4 are generic compound indices and $\bar{V}^{1234}$ is the Coulomb matrix element. Assuming low densities, the form of the Coulomb Hamiltonian that will mix different dipoles will have the following form,

$$H_C = \sum_{ss'\xi_\lambda\xi'_\lambda l_\lambda l'_\lambda \mathbf{kk'q}} \bar{V}^{CV}_{ss'\xi_\lambda\xi'_\lambda l_\lambda l'_\lambda}(\mathbf{k},\mathbf{k'},\mathbf{q}) v^\dagger_{\xi'_h s' l'_h,\mathbf{k'}-\mathbf{q}} c^\dagger_{\xi_e s l_e,\mathbf{k}+\mathbf{q}} c_{\xi'_e s' l'_e,\mathbf{k'}} v_{\xi_h s l_h,\mathbf{k}} \tag{3}$$

where $s^{(')} = (\uparrow,\downarrow)$ is the spin index, $\xi^{(')}_\lambda$ is the valley index and $l^{(')}_\lambda$ is the layer index with $\lambda =$ (c, v). The relative momenta is given by k and k' respectively, and the transferred momenta is given by q. Furthermore, $c^{(\dagger)}/v^{(\dagger)}$ are annihilation(creation) operators for the conduction band/valance band. Switching to the exciton basis, and only keeping the relevant mixing terms gives,

$$H_C = \sum_{\substack{\tilde{s}\tilde{s}'\xi\xi' LL' \\ \mathbf{kk'q}}} \bar{V}^{CV}_{\tilde{s}\tilde{s}',\xi\xi' LL'}(\mathbf{k},\mathbf{k'},\mathbf{q}) X^\dagger_{\xi\tilde{s}L,\mathbf{q}} X_{\xi'\tilde{s}'L',\mathbf{q}} \times \phi^{*\xi\tilde{s}l_e}_{\xi\tilde{s}l_h}(\mathbf{k}+\alpha^\xi\mathbf{q}) \phi^{\xi'\tilde{s}'l'_e}_{\xi'\tilde{s}'l'_h}(\mathbf{k'}-\beta^{\xi'}\mathbf{q}). \tag{4}$$

Here L = (l_e, l_h) is a compound layer index,s = ($\uparrow\uparrow, \downarrow\downarrow, \downarrow\uparrow, \uparrow\downarrow$) is a compound spin index (the spin triplet states will not be spin conserving and the matrix element will therefore be set to 0 for these). Note that in electron-hole picture, the spin for the hole would be reversed. The compound valley index is given by $\xi = (\xi_e, \xi_h)$ and $X^{(\dagger)}$ are exciton annihilation(creation) operators. Simplifying the above expression to only include the relevant terms for the quadropole coupling gives us

$$H_{QC} = \sum_{\tilde{s}\tilde{s}'\xi\xi' LL'\mathbf{Q}} \tilde{J}^{\tilde{s}\xi L}_{\tilde{s}'\xi' L'}(\mathbf{Q}) X^{\xi\tilde{s}\dagger}_{L\mathbf{Q}} X^{\xi'\tilde{s}'}_{L',\mathbf{Q}}, \tag{5}$$

where we have changed the matrix element to $\tilde{J}^{\tilde{s}\xi L}_{\tilde{s}'\xi' L'}(\mathbf{Q})$, which only includes the specific contributions of the Coulomb Hamiltonian that mixes different dipoles and the exciton wave functions. Moreover, we have changed notation of the transferred momentum so it directly corresponds to the center-of-mass momentum $\mathbf{Q}$. The matrix element $\tilde{J}^{\tilde{s}\xi L}_{\tilde{s}'\xi' L'}(\mathbf{Q})$ can thus be written out as

$$\tilde{J}^{\tilde{s}\xi L}_{\tilde{s}'\xi' L'}(\mathbf{Q}) = \sum_{\boldsymbol{k}'\boldsymbol{k}} \bar{V}^{CV}_{\tilde{s}\tilde{s}',\xi\xi' LL'}(\mathbf{k}, \mathbf{k'}, \mathbf{Q}) \phi^{*\xi\tilde{s}l_e}_{\xi\tilde{s}l_h}(\mathbf{k} + \alpha^{\xi}\mathbf{Q}) \phi^{\xi'\tilde{s}'l'_e}_{\xi'\tilde{s}'l'_h}(\mathbf{k'} - \beta^{\xi'}\mathbf{Q}). \tag{6}$$

The Coulomb matrix element can in turn be derived as[6]

$$\begin{aligned} \bar{V}^{CV}_{\tilde{s}\tilde{s}',\xi\xi' LL'}(\mathbf{k}, \mathbf{k'}, \mathbf{Q}) = \sum_{\boldsymbol{G}} \frac{V(\boldsymbol{G}+\boldsymbol{Q})}{A} \left\langle \mathcal{U}^{cl_e}_{\xi,\boldsymbol{k}+\boldsymbol{q},s}(\boldsymbol{r}) \middle| e^{i\boldsymbol{G}\cdot\boldsymbol{r}_1} \middle| \mathcal{U}^{vl_h}_{\xi,\boldsymbol{k},s}(\boldsymbol{r}) \right\rangle \\ \times \left\langle \mathcal{U}^{vl'_h}_{\xi',\boldsymbol{k}'-\boldsymbol{q},s'}(\boldsymbol{r}) \middle| e^{-i\boldsymbol{G}\cdot\boldsymbol{r}_1} \middle| \mathcal{U}^{cl'_e}_{\xi',\boldsymbol{k}',s'}(\boldsymbol{r}) \right\rangle, \end{aligned} \tag{7}$$

where $\mathcal{U}^{\lambda l_\lambda}_{\xi,\boldsymbol{k},s}(\boldsymbol{r})$ are the Bloch factors to the electronic Bloch wave function $\Psi^{\lambda l_\lambda}_{\xi,\boldsymbol{k},s}(\boldsymbol{r}) = e^{i(\boldsymbol{\xi}+\boldsymbol{k})\cdot\boldsymbol{r}} \mathcal{U}^{\lambda l_\lambda}_{\xi,\boldsymbol{k},s}(\boldsymbol{r})$ and $\boldsymbol{G}$ are the reciprocal vectors of the lattice.

As can be seen from Fig. S7 we have two distinct types of mixing between different dipoles. One case with the same valley, but different spin and one case with different spin, but the same valley. Consequently, we can rewrite the Hamiltonian into these two parts

$$H_{QC} = \sum_{\tilde{s}\tilde{s}'\xi\xi' LL'\mathbf{Q}} \left(J^{\tilde{s}\xi L}_{\tilde{s}'\xi L'}(\mathbf{Q}) \delta_{\xi\xi'} + J'^{\tilde{s}\xi L}_{\tilde{s}\xi' L'}(\mathbf{Q}) \delta_{\tilde{s}\tilde{s}'} \right) X^{\xi\tilde{s}\dagger}_{L\mathbf{Q}} X^{\xi'\tilde{s}'}_{L',\mathbf{Q}}. \tag{8}$$

Here, J and J' are the matrix elements for the two different forms of dipole exchange processes taken into consideration. For J', we have a mixing of different valleys, which consequently means we only have long-range interaction.[6] This corresponds to $\boldsymbol{G} = 0$ in

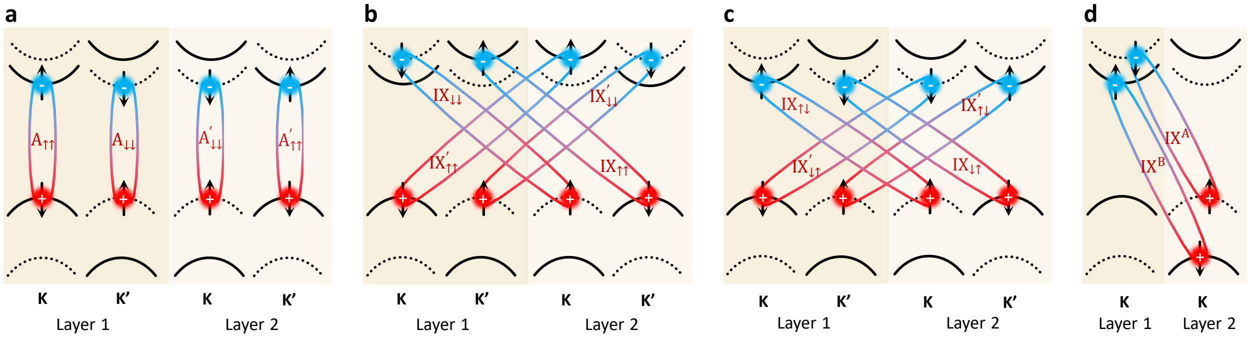

FIG. S7. **Excitonic states in $MoSe_2$ homobilayer.** Schematic showing the four degenerate states of A exciton **a**, spin-singlet IX **b**, spin-triplet IX **c**. When labelling the exciton state, the first arrow in the subscript denotes the spin of the electron and the second of the hole (which is reversed in the electron-hole picture). The prime superscript denotes a reversed dipole moment, which corresponds to switched position (layer) of the electron and the hole. **d** Example of the lower(higher) lying A-like (B-like) spin-singlet IX denoted with superscript "$^{A(B)}$", with the hole residing in higher(lower) valence band.

equation S7 and would be proportional to the optical dipole matrix element. The scenario is different for J were $\boldsymbol{G} \neq 0$ is also allowed due to the valleys being the same, this short-range addition to the term would then make $J > J'$. Matching these to experiments reveal that $J = 90$ meV and $J' = 8$ meV. These are much larger than the expected values, which should lie close to the value of the optical dipole matrix element, which for interlayer excitons are orders of magnitude smaller.

In our considered material, the situation becomes more complex than just two different interlayer excitons. In naturally stacked $MoSe_2$ we have four degenerate excitons. Two from layer degeneracy (K and K') and two from spin degeneracy, which can be seen for the four different A excitons (Fig. S1a). Due to one layer being stacked 180° with respect to the other (H-type stacking), the spin-orbit coupling can be considered as reversed in one layer. Therefore, the four degenerate bright A excitons stem from a combination of different valleys (K or K'), different spin and different layer. The same scenario happens for the bright spin-allowed interlayer exciton states (Fig. S1b). We have four degenerate interlayer excitons with different spin, valley configurations and layer index. Two of them will have the reversed dipole moment. The interlayer excitons and intralayer excitons can now couple to each other via electron or hole tunneling. Normally, electron tunneling is

symmetry forbidden in pure naturally stacked homobilayers.[7] The visible avoided crossing between the quadrupole excitons and the A exciton seen in the experiment suggests however that some limited electron tunneling is allowed. The carrier tunneling can be modeled in accordance with ref.[8] By including the new dipole exchange coupling we can now write down an extended Hamiltonian for field dependent exciton energy landscape

$$H = H_0 + H_T + H_{QC}, \tag{9}$$

where H_0 includes the bare exciton binding energies as obtained from the generalized Wannier equation[9] and the exciton response to the external electric field. The tunneling Hamiltonian is given by H_T, which takes into account both electron and hole tunneling. The last contribution is the new dipole exchange coupling giving rise to the formation of quadropole excitons. Writing out the full Hamiltonian gives us

$$\begin{aligned} H = \sum_{\xi\xi'\tilde{s}\tilde{s}'\mathbf{Q}LL'\mu\nu} \Bigg(& \Big(E^{\tilde{s}\xi\mu}_{L\mathbf{Q}} + d_L E_z\Big) X^{\mu\tilde{s}\xi\dagger}_{L\mathbf{Q}} X^{\mu\tilde{s}\xi}_{L\mathbf{Q}} \delta_{LL'}\delta_{\tilde{s}\tilde{s}'}\delta_{\xi\xi'}\delta_{\mu\nu} \\ & + T^{\mu\nu\xi}_{LL'} X^{\mu\tilde{s}\xi\dagger}_{L\mathbf{Q}} X^{\nu\tilde{s}\xi}_{L'\mathbf{Q}} \delta_{\tilde{s}\tilde{s}'}\delta_{\xi\xi'} + \Big(J^{\tilde{s}\xi L}_{\tilde{s}'\xi L'}(\mathbf{Q})\delta_{\xi\xi'} + J'^{\tilde{s}\xi L}_{\tilde{s}\xi' L'}(\mathbf{Q})\delta_{\tilde{s}\tilde{s}'}\Big) X^{\xi\tilde{s}\dagger}_{L\mathbf{Q}} X^{\xi'\tilde{s}'}_{L',\mathbf{Q}} \Bigg), \end{aligned} \tag{10}$$

where $E^{\tilde{s}\xi\mu}_{L\mathbf{Q}}$ is the bare exciton dispersion and $d_L E_z$ is the induced shift from the electrical field. Moreover, $T^{\mu\nu\xi}_{LL'}$ is the tunneling matrix element. Here, $\mu(\nu)$ are the exciton state indices summing over 1s and 2s. The dipole exchange coupling is given by J and J', where we have assumed that the coupling between 1s and 2s in J is small, thus only taking into account 1s for J/J'.

The complete Hamiltonian now gives rise to several different coupling channels. As an example the $\mathrm{IX}_{\uparrow\uparrow}$ exciton will couple to $\mathrm{IX'}_{\downarrow\downarrow}$ via the new coupling $\tilde{J}$, but each of the interlayer excitons will also couple to their respective A exciton via electron tunneling. Furthermore, they will also couple to the higher lying B exciton via hole tunneling. For the purpose of this effective model we have only considered a coupling between the four degenerate interlayer excitons in the coupling J/J'. Diagonalizing the Hamiltonian now gives us the final exciton energies.

In Fig. S8, the calculated exciton energies are shown as a function of electric field by including one coupling at a time. First, Fig. S8(a) shows the case in which only hole

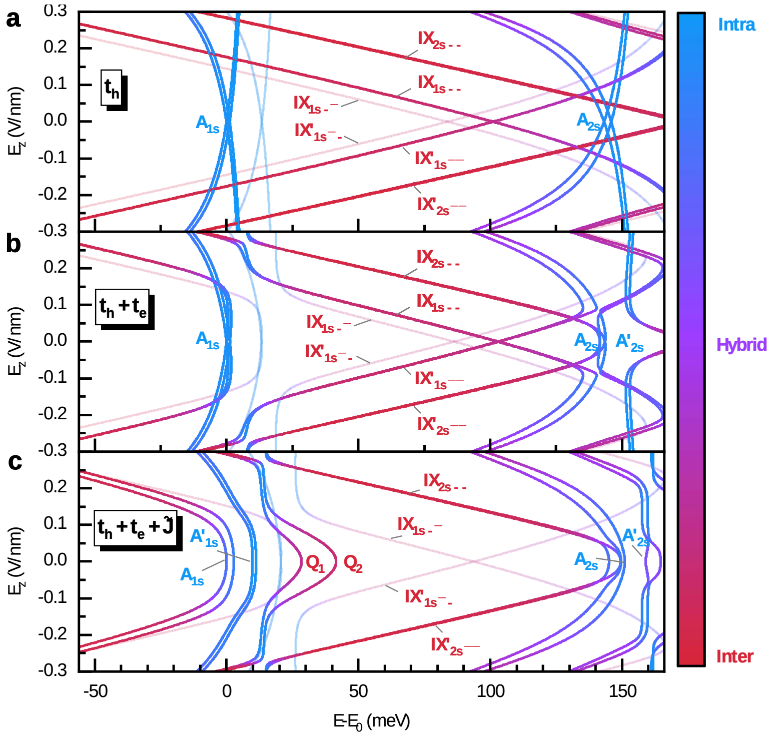

FIG. S8. **Simulated exciton energy landscape under the electric field with different coupling contributions.** Simulated evolution of the excitonic landscape in presence of the out of plane electric fields by inclusion of **a** hole tunneling, **b** hole and electron tunneling, **c** hole and electron tunneling as well as the new coupling dipole exchange coupling $\tilde{J} = J + J'$. The opaque and semi-transparent lines correspond to singlet and triplet states, respectively. The energy scale ($\mathrm{E}-\mathrm{E}_0$) is shown relative to the $\mathrm{A}_{1\mathrm{s}}$ exciton energy E_0.

tunneling is considered. Here, we can see multiple different shifts from the various interlayer excitons. Additionally the strong mixing of both A_{1s} and A_{2s} excitons with higher lying interlayer excitons due to hole tunneling results in their energy shift (splitting at higher electric fields) and change of the intra-inter layer character (See Fig. S4(c)) where all hole tunneling related hybridizations are marked). By then including electron tunneling (Fig. S8(b)) we can see the emergence of avoided crossings with the A exciton when the interlayer excitons pass through. Since the electron tunneling is much less efficient than the hole tunneling, the hybridization is far more localized around the crossing. Finally, by including the new couplings J/J' (Fig. S8(c)) we can see a clear emergence of quadrupolar excitons Q_1 and Q_2, exhibiting their characteristic parabolic shape. The higher lying quadrupoles are nearly completely suppressed due to tunneling with the 2s state, where we as a consequence

can see a splitting of the 2s state at 0 electrical field. We can also note that the 2s state will split at higher electrical field strengths due to efficient hybridization with the higher lying 2s interlayer exciton.

The splitting of the A exciton stems from a combination of hole tunneling and J/J'. Due to the hole tunneling with higher lying interlayer excitons, it will exhibit a very small, but non-zero interlayer character, which in turn means that it will be affected by the coupling J (same valleys, but different spin) and split some of the A excitons in an attempt to form quadrupoles. Similarly, a very small effect from J' (different valleys, same spins) can also be seen in the two lowest branches of the A exciton. Due to the weak hybridization of the A exciton, both effects are very much suppressed however. Note here, that all four A excitons are still mostly degenerate. The four different branches of the A exciton all exhibits a superposition of the original A exciton constituents and the splitting is only due to their very small interlayer nature. At larger electrical field strengths we can see the quadrupoles Q_1 and Q_2 cross the A exciton branches and via electron tunneling forming avoided crossings and then continuing to become the lower lying branches themselves. Here, we have also taken into account the spin triplet exciton states $IX_{\uparrow\downarrow}$ and $IX'_{\downarrow\uparrow}$. Since a coupling between these excitons would not be spin conserving it should not experience the coupling J. Therefore, the triplet interlayer exciton states follow the standard linear shift with electrical field instead.

III. ANALYSIS OF THE REFLECTIVITY SPECTRA

We show here briefly how the reflectivity spectra have been analyzed to obtain the false-color maps shown in the main article and in the Supplementary Information. In Fig. S9(a), we show a representative reflectivity spectrum R measured on the $MoSe_2$ bilayer and the reference spectrum R_0 used to normalize all the spectra, measured on the SiO_2 substrate. The choice of acquiring the reference spectrum on the SiO_2 substrate was dictated by the non-uniform hBN and graphene thickness in areas which did not include any TMD layer. Starting from the raw R and R_0, spectra their ratio was initially calculated, as shown in Fig. S9(b). After the first derivative with respect to the wavelength was computed and smoothed, see Fig. S9(c), a background subtraction, common for all the spectra measured as a function of the electric field in a specific spot, was performed, as illustrated in Fig. S9(d). Subsequently, the second derivative spectrum was calculated (derivation with respect to the wavelength), with the smoothing parameters specified in Fig. S9(e).

In Fig. S10 we show the comparison of the reflectivity spectra as a function of the electric field (positive direction) in the form of R/R_0, 1st derivative R/R_0 and 2nd derivative R/R_0 in panels (a,b,c), respectively. The quadratically shifting quadrupolar states Q_1 and Q_2 are only weakly visible on the raw R/R_0 spectra (panel (a)). Calculating the 1st (panel (b)) and then 2nd (panel (c)) derivative of the R/R_0 spectra substantially improves the visibility of these weak spectral features.

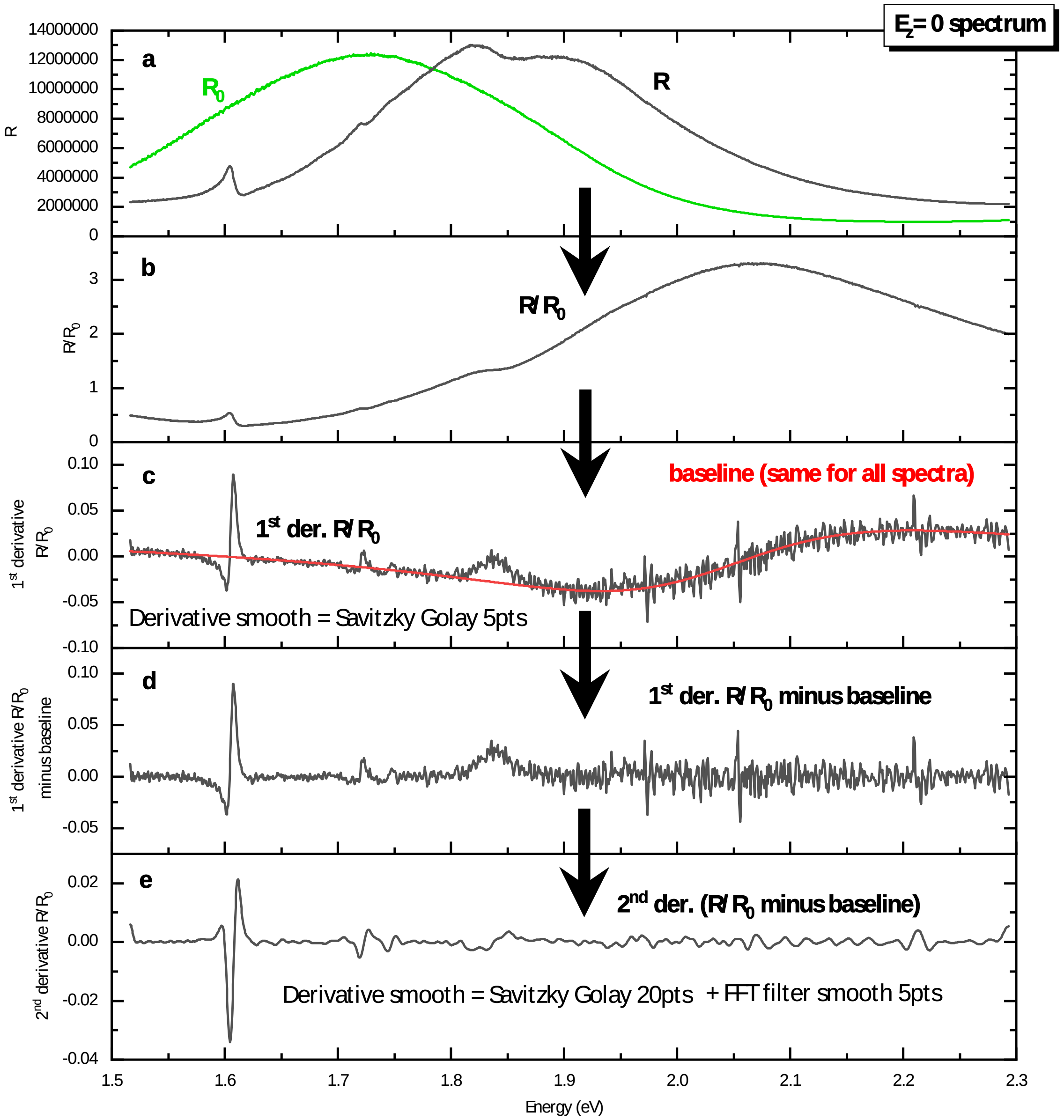

FIG. S9. **Analysis of the reflectivity spectra.** **a** Raw reflectivity spectrum R measured on the $MoSe_2$ bilayer and reference spectrum R_0 measured on the SiO_2 substrate. **b** Reflectivity ratio R/R_0 and **c** first derivative with respect to the wavelength. In red, the baseline of the derivative spectrum is shown. The smoothing parameters are indicated. **d** First derivative after the baseline subtraction. **e** Second derivative with the corresponding smoothing parameters.

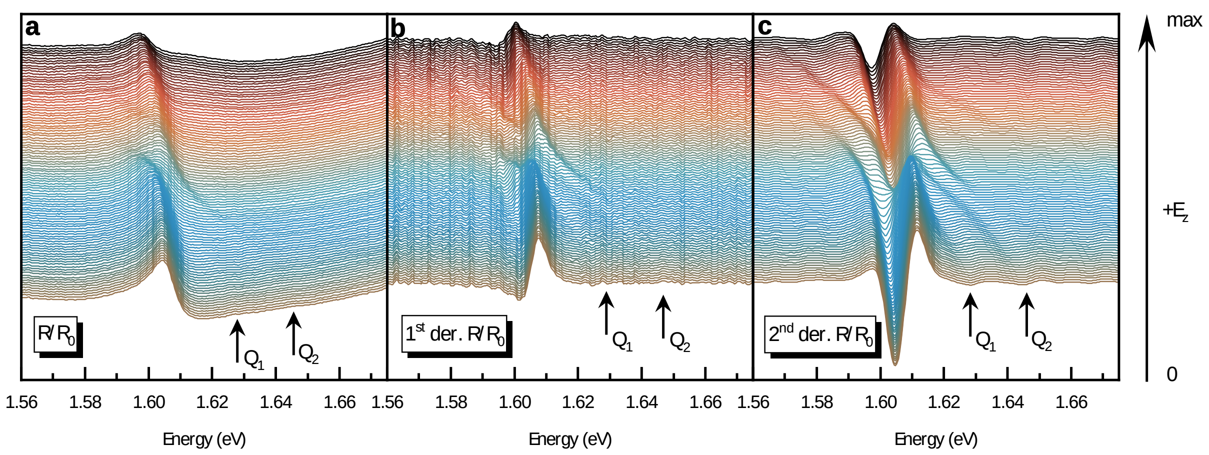

FIG. S10. **Reflectivity spectra under the electric field.** Vertically stacked reflectivity spectra as a function of E_z (positive direction) in the form of **a** R/R_0 **b** 1st derivative R/R_0 and **c** 2nd derivative R/R_0. The Q_1 and Q_2 transitions are marked on the zero-field spectra.

* paulina.plochocka@lncmi.cnrs.fr

[1] J. Sung, Y. Zhou, G. Scuri, V. Zólyomi, T. I. Andersen, H. Yoo, D. S. Wild, A. Y. Joe, R. J. Gelly, H. Heo, *et al.*, Nature nanotechnology **15**, 750 (2020).

[2] S. Kovalchuk, K. Greben, A. Kumar, S. Pessel, K. Watanabe, T. Taniguchi, D. Christiansen, M. Selig, A. Knorr, and K. Bolotin, arXiv preprint arXiv:2303.09931 (2023).

[3] V. Villafañe, M. Kremser, R. Hübner, M. M. Petrić, N. P. Wilson, A. V. Stier, K. Müller, M. Florian, A. Steinhoff, and J. J. Finley, Physical Review Letters **130**, 026901 (2023).

[4] J. Sell, J. Vannucci, D. Suárez-Forero, B. Cao, D. Session, H.-J. Chuang, K. McCreary, M. Rosenberger, B. Jonker, S. Mittal, *et al.*, Physical Review B **106**, L081409 (2022).

[5] M. Sidler, P. Back, O. Cotlet, A. Srivastava, T. Fink, M. Kroner, E. Demler, and A. Imamoglu, Nature Physics **13**, 255 (2017).

[6] H. Yu, G.-B. Liu, P. Gong, X. Xu, and W. Yao, Nature communications **5**, 3876 (2014).

[7] Y. Wang, Z. Wang, W. Yao, G.-B. Liu, and H. Yu, Phys. Rev. B **95**, 115429 (2017).

[8] J. Hagel, S. Brem, C. Linderälv, P. Erhart, and E. Malic, Phys. Rev. Res. **3**, 043217 (2021).

[9] S. Ovesen, S. Brem, C. Linderälv, M. Kuisma, T. Korn, P. Erhart, M. Selig, and E. Malic, Communications Physics **2**, 1 (2019).
